# Exercise Programs to Reduce the Risk of Musculoskeletal Injuries in Military Personnel: A Systematic Review and Meta‐Analysis

**DOI:** 10.1002/pmrj.12360

**Published:** 2020-04-22

**Authors:** Iris Dijksma, Ilgin G. Arslan, Faridi S. van Etten‐Jamaludin, Roy G. Elbers, Cees Lucas, Martijn M. Stuiver

**Affiliations:** ^1^ Clinical Epidemiology, Biostatistics and Bioinformatics, Master Evidence Based Practice in Health Care Amsterdam UMC, University of Amsterdam Amsterdam The Netherlands; ^2^ Defence Healthcare Organisation Ministry of Defence Utrecht The Netherlands; ^3^ Medical Library Amsterdam UMC, University of Amsterdam Amsterdam The Netherlands

## Abstract

**Objective:**

To evaluate the effect of exercise programs on reduction of musculoskeletal injury (MSI) risk in military populations.

**Design:**

Systematic review and meta‐analysis.

**Literature Survey:**

A database search was conducted in PubMed/MEDLINE, EMBASE, Cochrane Library, CINAHL, SPORTdiscus, WHO International Clinical Trials Registry Platform Search Portal, Open Gray, National Technical Reports Library, and reference lists of included articles up to July 2019. Randomized and cluster‐randomized controlled trials evaluating exercise programs as preventive interventions for MSIs in armed forces compared to other exercise programs or to usual practice were eligible for inclusion.

**Methodology:**

Two authors independently assessed risk of bias and extracted data. Data were adjusted for clustering if necessary and pooled using the random‐effects model when appropriate.

**Synthesis:**

We included 15 trials in this review, with a total number of 14 370 participants. None of the included trials appeared to be free of any risk of bias. Meta‐analysis and Grading of Recommendations, Assessment, Development and Evaluations (GRADE) assessment could be performed for static stretching compared to no stretching (3532 participants), showing low quality of evidence indicating no favorable effect of stretching. Gait retraining, an anterior knee‐pain targeted program, and resistance exercises showed cautious favorable effects on reducing injury risk in military personnel.

**Conclusion:**

The current evidence base for exercise‐based MSI prevention strategies in the military is of low quality. Areas worthy of further exploration include the effects of gait retraining, anterior knee‐pain targeted programs, agility training, and resistance training programs, on medial tibial stress syndrome incidence, anterior knee pain incidence, attrition due to injuries and any type of MSI, respectively.

## Introduction

Musculoskeletal injuries (MSIs) in military populations are a substantial problem. They reduce both training and operational effectiveness and increase the burden on associated medical care provision.^1^, ^2^, ^3^ Besides the resulting demand for health care and the personal impact of sustaining an MSI, the magnitude of the financial burden of MSIs on military budgets has also been recognized globally.^4^, ^5^, ^6^

MSIs comprise more than 150 diagnoses that affect the locomotor system—that is, muscles, bones, joints, and associated tissues such as tendons and ligaments. Such injuries are typically characterized by pain and limitations in mobility, dexterity, and functional ability.^7^ In the military, especially within units where daily tasks are physically demanding, pain and limited functionality of the locomotor system often result in exemption of military training and deployment abroad.^8^

The MSI rates of injuries in the military are equal or slightly higher compared to those among endurance athletes but quite lower than in contact sports.^4^, ^9^ Not surprisingly, MSIs are most seen in recruits and military trainees, compared to versed military personnel.^10^, ^11^ This is likely due to excessive and rapid increases in training loads in military trainees.^12^ Intending to build robust and “unbreakable” soldiers, building resilience to training load is fundamental.^13^ In 2016, Gabbett^14^ proposed the training‐injury prevention paradox: Physically hard training develops physical qualities, which develop greater resilience and training tolerance, which in turn *protect* against injuries. Gabbett has written that “high training workloads alone do not cause sports injuries: how you get there is the real issue.” In addition, Drew and Purdam proposed use of the term “*training load error*” instead of overuse injuries.^15^ Although variables such as training load and volume are parts of the web of determinants, they are also highly modifiable, and therefore they are of interest for prevention strategies in the military.^16^, ^17^

Several studies have been done to estimate the effects of interventions aiming to reduce the risk of MSIs in sports and military populations. This includes modifications to equipment and supplementation (ie, insoles,^18^ specific footwear,^19^, ^20^ post‐exercise protein supplementation^21^) as well as modifications to training and exercise programs (ie, neuromuscular training and resistance exercises).^2^, ^22^, ^23^, ^24^ To date, recommended strategies to reduce the risk of MSIs in military personnel include prevention of overtraining, performance of neuromuscular training, awareness of injury prevention by individuals in leadership positions, and improving physical fitness in the absence of excessive time on foot to reduce MSI rates.^3^, ^25^ Specifically, regarding the ambition to prevent training‐related injuries, vigorous collaboration across commands, operators, researchers, health care providers, sports instructors, and training commands to institutionalize current best practices is of critical importance.^12^ However, identification of best practices is hindered because systematic reviews synthesizing the evidence for MSI‐prevention interventions based on rigorous searches and appraisal of methodological validity are currently lacking, as all available reviews are of a narrative or scoping nature.^3^, ^25^ As a result, studies may be missed in those reviews and biases in original studies are possibly ignored in formulating recommendations. Therefore, we conducted a systematic review, restricted to randomized controlled trials evaluating exercise programs, to provide the best possible evidence‐based recommendations.

With the aim to improve policymaking concerning MSI prevention in the military, we performed a systematic review to assess the effectiveness of (adjustments to) training programs for preventing acute and training‐related MSIs in armed forces. This review closely follows the recommendations of the Cochrane Handbook for Systematic Reviews.^26^ It includes a critical appraisal of the included studies through a validated system, meta‐analyses, and a summary of findings via the Grading of Recommendations, Assessment, Development and Evaluations (GRADE) system.^27^

## Methodology

This systematic review was performed and reported using the guidelines provided by the Preferred Reporting Items for Systematic Review and Meta‐Analyses (PRISMA) statement.^28^ The review protocol was registered in PROSPERO (CRD42017062208).^29^ We conducted a search in PubMed/MEDLINE, EMBASE (Ovid), Cochrane Library, CINAHL (Ebsco) and SPORTdiscus (Ebsco) up to July fifth, 2019 (see online Appendix A). We searched the World Health Organization International Clinical Trials Registry Platform Search Portal, Open Gray, and National Technical Reports Library, to identify ongoing, recently completed, and unpublished studies. Lastly, we identified additional records through searching the reference lists of included articles.

### *Inclusion Criteria for Studies*

The following eligibility criteria for studies to be included in this review were used. Design: randomized controlled trial (RCT) or cluster‐RCT; Population: military personnel in active service or recruits in military training, of either gender, rank or occupational function and adolescence to middle age (60 y); Intervention: exercise programs compared with another exercise program or usual practice; Primary outcome: number of participants sustaining any type of MSIs and/or incidence of any type of MSIs and/or withdrawals due to any type of MSIs as primary or secondary outcome; Secondary outcomes: limited duty days and compliance to the intervention. Injuries could be self‐reported or diagnosed by a medical practitioner. Conference abstracts were excluded, as were studies written in languages other than English or Dutch. Also, a review of MSI preventive interventions other than exercise programs are reported in a separate article.

### *Outcome Measures of the Review*

Outcomes for this study were any type of MSI incidence rates, withdrawals due to MSIs, limited‐duty days due to MSIs, and compliance to the intervention. Interventions were divided into two broad categories: preventive programs and modification of training programs. Subgroup analyses within these categories were planned if appropriate to explore the impact of clinical heterogeneity within these categories.

### *Study Selection and Data Extraction*

Two review authors (I.D., I.A.) independently screened the titles and abstracts of identified records for in‐and exclusion criteria and examined full‐text versions of potentially eligible articles. Review authors were not blinded to authors of the papers or to the institutions commissioning or conducting the studies. The same review authors (I.D., I.A.) independently extracted data using a pretested data extraction form based on the template provided in the Cochrane Handbook.^26^ Data extraction forms were compared after completion and inconsistencies were solved by consensus and if necessary by scrutiny from the last review author (M.S.).

### *Risk of Bias Assessment*

Risk of bias in the included studies was independently assessed by two review authors (I.D., I.A.) using the Cochrane's Risk of Bias tool and following the recommendations of the Cochrane Handbook Version 5.1.0.^26^ For cluster‐RCTs, explicit consideration was given to inappropriate analyses. Not accounting for clustering and/or lack of adjustment for imbalanced covariates were considered as an unclear and high risk of other bias, respectively. For all studies, not accounting for dependent observations while reporting number of MSIs (ie, not applying multilevel analysis when number of injuries was reported instead of number of participants sustaining one or more MSIs), differential treatment besides the intervention or follow‐up time, and deviation from the study protocol were also considered as high risk of (other) bias. Disagreement was resolved by consensus if necessary followed by scrutiny from the last author (M.S.).

### *Data Analysis*

All analyses were done in Review Manager (RevMan) V.5.3.^30^ Two by two tables were reconstructed if possible based on the reported number of events and the number of analyzed participants in each group. When studies were considered clinically homogeneous, statistical pooling was attempted using the generic‐inverse variance method with relative risk ratios (RR) and a random‐effects model in RevMan. This method applies an inflated SE that accounts for the clustering.^26^ Intervention effects were expressed in RR or hazard ratio (HR) including 95% confidence intervals (CI). In the case of clinical heterogeneity, we did not pool the results. When studies were clinically homogeneous, residual statistical heterogeneity between the studies was checked by visual inspection of the forest plots, Q‐test, and I^2^ statistics.^31^ An I^2^ below 50% was considered sufficient homogenous for pooling the results. In case of missing data due to insufficient reporting, we contacted corresponding authors of included studies. If missing data could not be retrieved, and two by two tables could not be constructed, a narrative summary of the reported outcomes was provided instead.

We used the GRADE‐approach to define the quality of evidence for each pooled outcome.^27^, ^31^, ^32^, ^33^, ^34^, ^35^ GRADE provides an overall summary of the quality of the evidence for each individual outcome, considering methodological quality of the studies including that outcome, as well as uncertainty related to imprecision or heterogeneity. For this review, the starting grade of quality of evidence was “high quality,” as the results were obtained from RCTs and cluster‐RCTs. Downgrading of the quality was performed using the criteria of the GRADE‐system, and reasons for downgrading are reported.^27^

## Results

**Figure 1:**
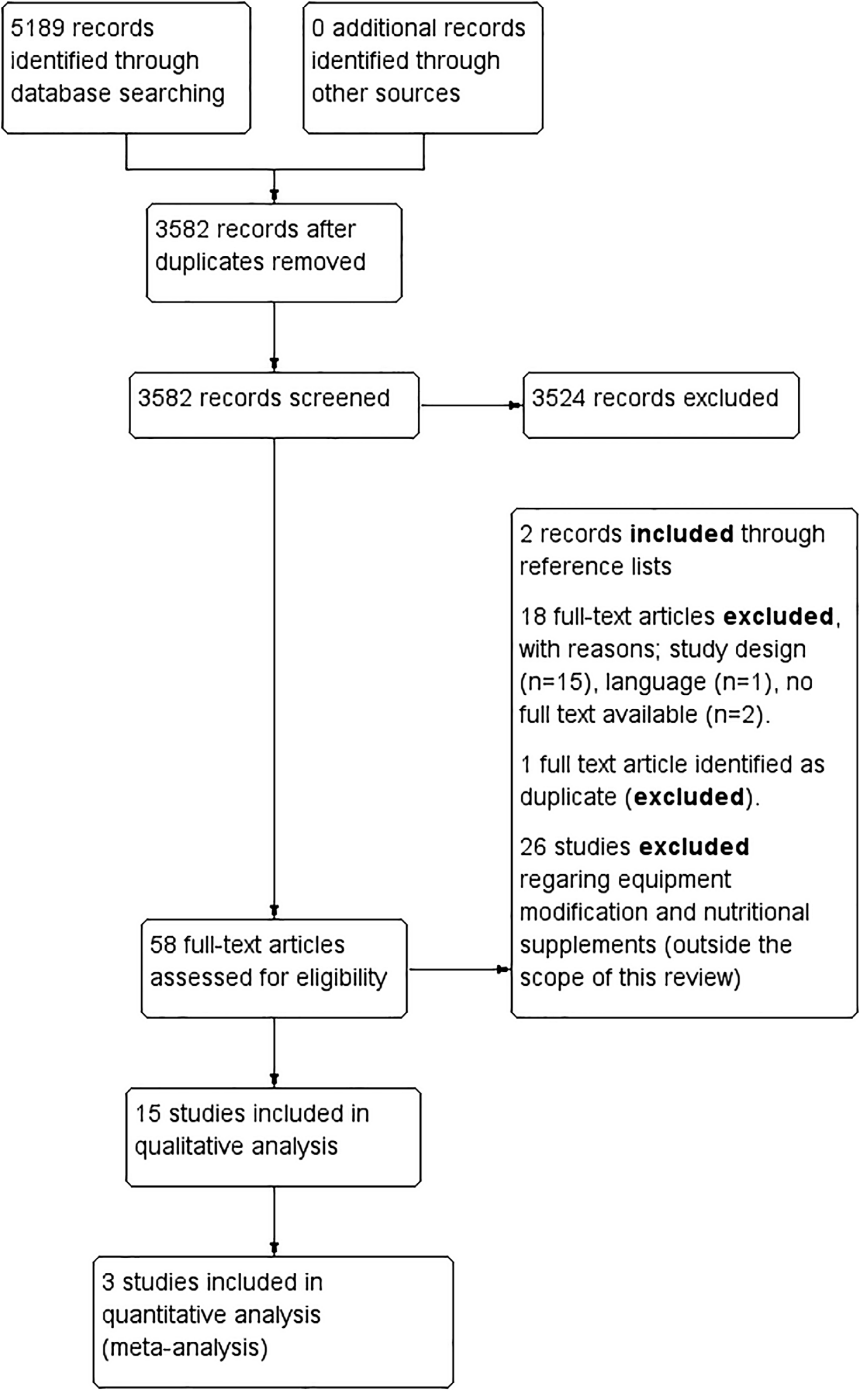
Flowchart of the inclusion and exclusion of articles in this review.

The search identified 5189 records. After removing duplicates, a total of 3582 articles were left and screened on title and abstract, of which 3524 articles were excluded. The remaining 60 articles were screened on full texts and of those 15 trials were included for this review. Thirteen of the included trials were cluster‐RCTs and two trials were individually randomized RCTs.^36^, ^37^ Flowchart of studies is presented in Figure 1, and the main reasons for exclusions of full‐texts are detailed in online Appendix B.

The age of participants ranged from 16‐50 years, and they were employed as officer cadets, military recruits, and military personnel in active duty. Most studies involved only male participants, but four studies also included female participants (ranging from 10% to 40% female).^38^, ^39^, ^40^, ^41^ Two studies did not report on gender.^42^, ^43^ Study details are presented in detail in online Appendix C.

Due to clinical heterogeneity, we had limited opportunities to pool data. Meta‐analysis could be performed only for preventive stretching compared to no stretching.

Original data could not be obtained for one study, despite efforts to request additional data from the first author of the study.^37^

### *Risk of Bias*

**Figure 2:**
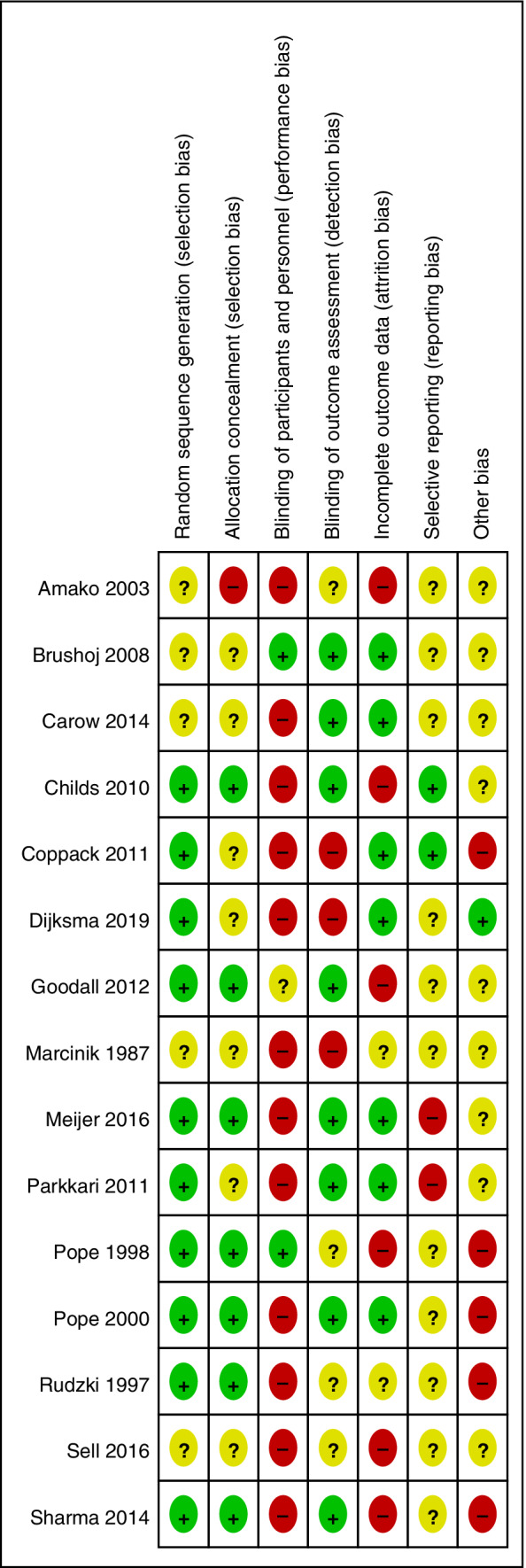
Risk of bias summary: review authors' judgments about each risk of bias item for each included study. Legend: + is low risk of bias, − is high risk of bias,? is unclear risk of bias.

No trials were deemed to be free of any risk of bias. Risk of bias was often judged as unclear due to insufficient reporting. Reasons for high risk of bias included the absence of blinding of participants and personnel for group assignment in many studies and attrition bias. Full details of the risk of bias assessment for each trial, including justification, are presented in online Appendix C. Figure 2 presents a risk of bias summary including review authors' judgments about each risk of bias item for each included study.

#### Preventive Programs

**Figure 3:**
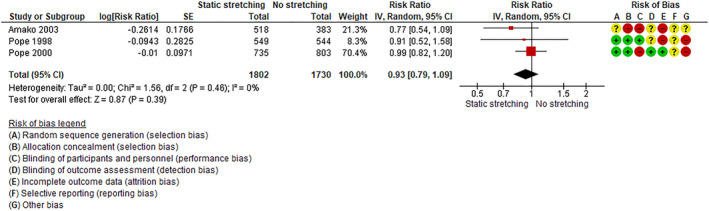
Forest plot of static stretching vs no‐stretching. Legend: + is low risk of bias, − is high risk of bias,? is unclear risk of bias.

Three trials investigated the effect of static stretching compared to no stretching on all types of MSIs (eg, bone, joint, muscle/tendon, spinal injuries)^44^ and lower limb injuries, with moderate risk of bias.^45^, ^46^ The pooled estimate showed a small and nonsignificant effect on total injury risk (see Figure 3: I^2^ = 0%, RR = 0.93, 95% CI 0.79‐1.09). Although stretching protocols differed, none of the trials individually provided evidence to support static stretching prior to exercising or stretching regularly outside of exercising for preventing all types MSIs. However, one of the trials^44^ concluded that static stretching may reduce the risk of muscle/tendon related and overuse injuries.

### *GRADE*

We assessed the overall quality of evidence of the comparison preventive stretching versus no stretching. The current evidence provides low quality of evidence that static stretching does not reduce the risk of musculoskeletal injuries (see Table 1, “Summary of findings”).

**Table 1:** Summary of findings

Static stretching compared to no stretching for preventing musculoskeletal injuries in armed forces
Patient or population: preventing musculoskeletal injuries in armed forces Setting: military setting involving officer cadets, military recruits, and military personnel in active duty Intervention: static stretching Comparison: no stretching

Outcomes	**Anticipated absolute effects** * (95% CI)		Relative effect (95% CI)	№ of participants (studies)	Certainty of the evidence (GRADE)	Comments
	**Risk with no stretching**	**Risk with static stretching**				
All types of musculoskeletal injuries	147 per 1.000	**137 per 1.000** (116 to 161)	RR 0.93 (0.79 to 1.09)	3532 (3 RCTs)	⊕⊕○○LOW † ^,^ ‡	Static stretching may not reduce the risk of all types of musculoskeletal injuries in military personnel.

GRADE Working Group grades of evidence
High certainty: We are very confident that the true effect lies close to that of the estimate of the effect
Moderate certainty: We are moderately confident in the effect estimate: The true effect is likely to be close to the estimate of the effect, but there is a possibility that it is substantially different
Low certainty: Our confidence in the effect estimate is limited: The true effect may be substantially different from the estimate of the effect
Very low certainty: We have very little confidence in the effect estimate: The true effect is likely to be substantially different from the estimate of effect

CI, confidence interval; RR, risk ratio.

* The risk in the intervention group (and its 95% confidence interval) is based on the assumed risk in the comparison group and the relative effect of the intervention (and its 95% CI).

Explanations:

† Several items of the risk of bias assessment were judged as unclear or high risk of bias.

‡ 95% Confidence interval overlaps no effect.

#### Modification of Training Programs

Twelve trials examined the effect of a modified training schedule on preventing different types of MSIs. Due to clinical heterogeneity of the interventions, pooling was not possible. Specific modified training programs are listed below and presented in a table in online Appendix D.

### *Core Stability*

Two trials^39^, ^47^ investigated the effects of core stability exercises programs compared to traditional exercise programs. Of these, one unpublished internal report^47^ (n = 252) showed no reduction of nontraumatic MSIs by adding core stability exercises to the regular physical training program (RR = 1.04, 95% CI 0.78‐1.38). The other trial^39^ also found no risk reduction of any type of MSI of a core stabilization exercise program without sit‐up training compared to a traditional exercise program with sit‐up training (RR = 1.09, 95% CI 0.96‐1.24).

Both trials reported no statistically significant or clinically relevant difference in withdrawal from training (RR = 1.04, 95% CI 0.82‐1.33)^47^ or limited duty days (a mean decrease of 1 day in the intervention group, *P* = .919).^39^ Both trials were considered to have low risk of bias on most criteria.

### *Gait Retraining*

One trial^37^ investigated the effect of a supervised gait retraining program in combination with neuromuscular and strengthening exercises compared to usual military training on medial tibial stress syndrome (MTSS) incidence in recruits. The intervention had a large effect and significantly reduced MTSS incidence rates (HR = 0.25, 95% CI 0.05‐0.53), but with a high risk of bias due to performance bias, attrition bias, and nonspecific effects due to other (neuromuscular) exercises besides the gait retraining.

### *Running Substituted by Marching*

One trial^48^ investigated the effect of substitution of running training by weighted marching (Walk), compared to usual practice (Run). Usual practice included 26.5 km running and 81 km marching, in a 12‐week recruit training course. Primary outcome of the study was the number of injured recruits. The risk of MSIs was higher in the Run group compared to the Walk group (RR = 1.24, 95% CI 0.98‐1.61). Risks of number of lower limbs injured and the number of knee injuries were RR = 1.65 95% CI 1.21‐2.25, and RR = 2.14, 95% CI 1.21‐3.79, respectively. The study had an unclear risk of bias on most criteria due to insufficient reporting.

### *Movement Enhancement Warm‐Up*

One trial^38^ explored the effects of a dynamic integrated movement enhancement warm‐up, compared to an active warm‐up, before engaging in sports or other intense physical training on the incidence of lower extremity injuries. This study found no difference between the groups (RR = 1.02, 95% CI 0.85‐1.22). Risk of bias was unclear on most criteria but low on detection bias and attrition bias.

### *Anterior Knee Pain Prevention Program*

One trial^40^ investigated the effects of a training program aimed at the prevention of anterior knee pain (AKP) in recruits. The program included bodyweight strengthening exercises for the lower extremities and static stretches. The intervention was compared to common warm‐up and warm‐down exercises as slow running, general stretching, abdominal curls, and push‐up drills. The AKP prevention training program strongly reduced both the risk of AKP (RR = 0.27, 95% CI 0.14‐0.54), as well as discharges for medical reasons (RR = 0.12, 95% CI 0.04‐0.39). However, this trial had a high risk of bias due to lack of blinding of participants and outcome assessors and a different follow‐up time in the two study arms. The AKP prevention program had a high compliance rate of 91%.

### *Neuromuscular Body Weight Exercises*

Three trials^41^, ^42^, ^49^ investigated the effects of neuromuscular bodyweight exercises for prevention of MSIs, and one trial^50^ investigated the effect of agility training on injury attrition rates in recruits undergoing 23 week initial military training.

One trial^41^ examined the effects of balance and agility training *as an extra training element* to the basic training program of army recruits, on lower limb injury incidence. The authors concluded that adding such exercises was possibly harmful and trainers and commanders must be cautioned (RR = 1.25, 95% CI 0.97‐1.53).

One study^42^ investigated the effect of an exercise program with muscular strengthening, coordination, and flexibility, targeting identified intrinsic risk factors, on overuse injury to the knee or shin. This showed no significant differences compared to a placebo training program (RR = 1.05, 95% CI 0.98‐1.11). Compliance to the intervention was 75%.

Neuromuscular and balance training with injury prevention counseling^49^ appeared to decrease the risk of acute ankle injuries (HR = 0.34, 95% CI 0.15‐0.78), and recruits tended to have less time lost due to injuries (HR = 0.55, 95% CI 0.29‐1.04).^49^ Compliance rate to the intervention was high at 83%.

One trial^50^ investigated the effects of three times per week a substitution of 20 minutes of the usual physical training program by agility training on attrition due to injuries in recruits compared to usual practice. This study did not report on incidence rates of MSIs; however, the authors found that agility training may reduce attrition due to injuries (RR = 0.32, 95% CI 0.12‐0.85). Compliance to the intervention was 72%.

Risk of biases were mostly judged at low and unclear, except for blinding of participants and personnel, which was judged as high risk of bias due to no blinding in two studies.^49^, ^50^

### *Resistance Training*

One trial^36^ included circuit weight training consisting of 15 exercises three times per week, compared to calisthenics exercises, both followed by an endurance run, on sprain and strain injury incidence in recruits.^36^ This resulted in a significantly reduced injury risk (RR = 0.82, 95% CI 0.72‐0.93), and a decrease of the number of no march/no physical training days lost on account of injury (123 d in the intervention group vs 330 d in the control group, *P* < .05).

One other trial^43^ described the effects of a program including speed/agility/balance, muscular strength, interval running, power, and endurance training, compared to control intervention which consisted of cardiorespiratory or strength activities as sandbag circuits on the risk of any type of MSI in soldiers.^43^ This trial also showed a reduction of MSI risk in the intervention group (RR = 0.66, 95% CI 0.47‐0.93). Both studies had an unclear to high risk of bias mostly due to insufficient reporting.^36^, ^43^

## Discussion

This systematic review provides an overview of the current evidence regarding preventive interventions for MSis in armed forces. The current evidence base for exercise programs as prevention strategies for MSIs in the military is of low quality. Promising areas worthy of further exploration include gait retraining, anterior knee‐pain targeted programs, agility training, and resistance training programs, on MTSS incidence, AKP incidence, attrition due to injuries, and any type of MSI, respectively.

We aimed to expand the work of our colleagues^3^, ^25^ by specifically addressing their study limitations—namely a systematic literature search, random allocation, assessing risk of bias of included studies—in our systematic review, and by differentiating between different types of exercise programs. Overall, the risk of bias of the included studies was considerable. Only 36% of judgments per item were “low risk of bias”. Thirty‐seven percent of all judgments were considered “unclear risk of bias,” mostly because of insufficiently detailed reporting. As in any pragmatic intervention, blinding of participants for exercise interventions is a challenge and often impossible. As a result, in 12 out of 15 studies we considered this item as “high risk of bias.” Although this is largely unavoidable, it does reduce the quality of the evidence. Detection bias due to knowledge of the allocated intervention could occur if participants were more likely to report their MSIs or visit a military physician for an MSI knowing they were enrolled in an experimental intervention. Most studies were designed to ensure that outcome assessors were blinded, to lower the chance of detection bias. Attrition bias was considerable in most studies, due to the high dropout rate that is typical for (basic) military training. Most studies lacked a detailed description of loss to follow‐up and statistical methods of handling missing data. Therefore we were unable to judge the impact and direction of bias. This could be improved in future studies. Also, very few study protocols were published a priori, which hindered the assessment of selective reporting. Prior registration of trials and/or publishing of design papers would improve transparency and should be encouraged for future trials on MSI prevention in the military. Finally, in cluster‐RCTs and repeated measures designs, observations tend to be more alike than entirely independent observations, for that reason adjustments for clustering by performing a multilevel analysis need to be carried out to adjust for clustering of the data.^51^ Only one study that we included in this review reported that adjustments of the results for clustering of the data has been carried out.^40^

Although sound experimental evaluations in military populations are lacking, our findings are in alignment with the previously described beneficial relationship between proprioceptive—and tissue strengthening resistance exercises and *lowering* the risk of MSIs.^52^ Because etiological factors of MSIs in military populations suggested in the literature include low body mass, low fitness, and previous injuries,^4^, ^11^ we hypothesize that the beneficial effects of resistance—and neuromuscular training programs on MSIs are mediated by an increase in physical fitness, load capacity, coordination, power endurance, and proprioception.^52^, ^53^ In sports science, there is evidence that neuromuscular training programs (ie, proprioceptive, agility, plyometrics) are effective for reducing the risk of (sports) MSIs in general.^23^, ^54^

It is worth mentioning that exercise programs designed to reduce the risk of particular MSIs—MTSS,^37^ AKP^55^—seem promising in reducing injury risk in military personnel. Because different MSIs have different risk factors, and therefore different factors to target in preventive interventions, specifying exercise programs seems valuable.^4^ Also, the mechanisms and controllability of acute injuries compared to training‐related injuries differ.^52^ One study in this review reported specifically that neuromuscular and balance training with injury prevention counseling decreased the risk of acute ankle injuries.^49^ To increase consciousness and knowledge concerning the prevention of specific MSIs, detailed description of outcome measures in future studies are recommended.

Despite our conclusion that static stretching does not appear to reduce the risk of any types of MSIs, we do believe that dynamic mobility workouts have a place in recovery protocols. After training and in between sessions, dynamic mobility workouts can help restore full range of motion and reduce perceived delayed onset muscle soreness.^56^

Unfortunately, very few studies reported compliance rates to the allocated intervention. However, those who did reported very high compliance rates. This is likely due in part to the military—hierarchical—setting, where strict supervision and mandatory training programs create a structured environment to investigate the effects of preventive exercise programs. Studies that reported protocol violations reported that missed training sessions were mostly due to field exercises.^42^, ^50^ In basic military training, physical activity is not restricted to sports training sessions but also includes weighted marches, contact drills, and maneuvering through urban terrain.

Notably, the two studies^41^, ^47^ that reported a possible harmful effect of the intervention noted that the intervention—balance and agility training and core stability training respectively—were *extra training requirements in the intervention group*. Nothing was removed from the training programs to compensate for this addition. Furthermore, previous studies have recommended both improving physical fitness and reducing physical activity volume as injury prevention strategies.^3^ We would emphasize that preventing training load error, by conscious load management and training harder and smarter, could be an effective injury prevention strategy in military personnel.^13^, ^14^ However, research regarding load management and injury risk in military populations is necessary to substantiate this.

For the sake of transparency, we registered this systematic review a priori in PROSPERO.^29^ We attempted to minimize publication bias by carrying out a sensitive search in several databases. Also, we minimized bias in the review process by having two authors independently screening studies for inclusion, extracting data, and assessing risk of bias. Yet, this review also has some limitations. First, type and definition of MSIs varied across studies, which made the effects of the included trials less comparable. Second, our findings concern predominantly male participants; effectiveness in female populations is also yet to be confirmed.^57^ Finally, due to clinical heterogeneity, we chose not to pool results other than static stretching interventions. Pooling would have been inappropriate, but as a consequence this review lacks data aggregation regarding adjustments to exercise programs.

Summarizing, we conclude that there is low quality evidence regarding preventive interventions for MSIs in armed forces. More well‐designed RCTs are needed to provide high‐quality recommendations. Trials involving more variation in gender are preferable in order to increase the generalizability of the results. Also, more adequate reporting of trial methods and results are needed to facilitate a complete overview of the risk of bias and to enable meta‐analysis for future systematic reviews.

## Practical Implications

Our recommendations for practice are (1) adaptation of neuromuscular and resistance exercises to strengthen tissues and increase fitness in both recruits as military personnel in active duty, (2) diagnosis‐specific targeted training programs, and (3) prevention of overly high acute loads and appropriate recovery time in between training sessions.

## Supporting information


**Appendix** **S1**: SUPPORTING INFORMATION
