# Supplementary material for: Exercise Programs to Reduce the Risk of Musculoskeletal Injuries in Military Personnel: A Systematic Review and Meta‐Analysis
Source: PM R. 2020 Apr 22;12(10):1028–37. doi: 10.1002/pmrj.12360 (PMC7586796; doi:10.1002/pmrj.12360)
Supplement: Supplementary file 1 — Appendix S1: SUPPORTING INFORMATION [file PMRJ-12-1028-s001.docx]

**Appendices**

Appendix A: electronic search strategies

**PubMed:**

(("Military Personnel"[Mesh] OR "Military Medicine"[Mesh] OR "Naval Medicine"[Mesh] OR militar*[tiab] OR armed force*[tiab] OR air force*[tiab] OR army[tiab] OR armies[tiab] OR infantr*[tiab] OR soldier*[tiab] OR warfighter*[tiab] OR naval[tiab] OR navy[tiab]) AND ("Musculoskeletal System"[Mesh] OR "Muscle, Skeletal/injuries"[Mesh] OR "Muscles/injuries"[Mesh] OR "Musculoskeletal Pain"[Mesh] OR "Sprains and Strains"[Mesh] OR "Athletic Injuries"[Mesh] OR "Leg Injuries"[Mesh] OR "Hip Injuries"[Mesh] OR "Extremities/injuries"[Mesh] OR "Arm Injuries"[Mesh] OR "Shoulder Injuries"[Mesh] OR "Back Injuries"[Mesh] OR "Soft Tissue Injuries"[Mesh] OR (("Exercise"[Mesh] OR "Sports"[Mesh]) AND injur*[tiab]) OR "Track and Field"[Mesh] OR musculoskeletal[tiab] OR musculo-skeletal[tiab] OR overus*[tiab] OR overtrain*[tiab] OR overexertion*[tiab] OR over-exertion*[tiab] OR overstrain*[tiab] OR excessive training[tiab] OR skeletal muscle injur*[tiab] OR leg injur*[tiab] OR knee injur*[tiab] OR ankle injur*[tiab] OR foot injur*[tiab] OR hip injur*[tiab] OR arm injur*[tiab] OR shoulder injur*[tiab] OR back injur*[tiab] OR soft tissue injur*[tiab] OR sprain*[tiab] OR strain*[tiab] OR cumulative trauma[tiab] OR muscle injur*[tiab] OR bone injur*[tiab] OR joint injur*[tiab]) AND ("Wounds and Injuries"[Mesh] OR "injuries" [Subheading] OR injur*[tiab] OR trauma*[tiab] OR rupture*[tiab] OR fracture*[tiab]) AND ("Physical Education and Training"[Mesh] OR "prevention and control" [Subheading] OR "Accident Prevention"[Mesh] OR "Preventive Medicine"[Mesh] OR prevent*[tiab] OR stretch*[tiab] OR training[tiab] OR protect*[tiab] OR precaution*[tiab])) NOT ("Animals"[Mesh] NOT "Humans"[Mesh])

**EMBASE (Ovid)**

Database(s): Embase Classic+Embase 1947 to 2017 January 31 
Search Strategy:

| # | Searches |
| --- | --- |
| 1 | soldier/ or military medicine/ or exp military phenomena/ or (militar* or armed force* or air force* or army or armies or infantr* or soldier* or warfighter* or naval or navy).ti,ab,kw. |
| 2 | exp musculoskeletal system/ or exp musculoskeletal pain/ or exp musculoskeletal injury/ or exp sprain/ or muscle strain/ or exp cumulative trauma disorder/ or sport injury/ or exp leg injury/ or exp limb/ or exp arm injury/ or shoulder injury/ or soft tissue injury/ or (musculoskeletal or musculo-skeletal or overus* or overtrain* or overexertion* or over-exertion* or overstrain* or excessive training or cumulative trauma or sprain* or strain*).ti,ab,kw. |
| 3 | (exercise/ or exp sport/ or fitness/) and injur*.ti,ab,kw. |
| 4 | ((skeletal muscle* or leg* or knee* or ankle* or foot or feet or hip* or shoulder* or back* or soft tissue* or muscle* or bone* or joint*) adj3 injur*).ti,ab,kw. |
| 5 | 2 or 3 or 4 |
| 6 | exp injury/ or (injur* or trauma* or rupture* or fracture*).ti,ab,kw. |
| 7 | physical education/ or prevention.fs. or prevention/ or "prevention and control"/ or accident prevention/ or preventive medicine/ or (prevent* or stretch* or training or protect* or precaution*).ti,ab,kw. |
| 8 | 1 and 5 and 6 and 7 |
| 9 | animal/ not human/ |
| 10 | 8 not 9 |

**CINAHL (Ebsco):**

(MH "Military Personnel+") OR (MH "Military Medicine") OR ( TI ( militar* or armed force* or air force* or army or armies or infantr* or soldier* or warfighter* or naval or navy ) OR AB ( militar* or armed force* or air force* or army or armies or infantr* or soldier* or warfighter* or naval or navy ) ) AND ( (MH "Musculoskeletal System+") OR ( (MH "Sprains and Strains+") ) OR (MH "Athletic Injuries+") OR (MH "Leg Injuries+") OR (MH "Extremities+/IN") OR (MH "Arm Injuries+") OR (MH "Back Injuries+") OR (MH "Soft Tissue Injuries+") OR ( (MH "Track and Field") ) OR ( TI ( musculoskeletal or musculo-skeletal or overus* or overtrain* or overexertion* or over-exertion* or overstrain* or excessive training or cumulative trauma or sprain* or strain* ) OR AB ( musculoskeletal or musculo-skeletal or overus* or overtrain* or overexertion* or over-exertion* or overstrain* or excessive training or cumulative trauma or sprain* or strain*) ) OR ( (MH "Exercise+") AND ( TI injur* OR AB injur* ) ) OR ( (MH "Sports+") AND ( TI injur* OR AB injur* ) ) ) OR ( (TI ( skeletal muscle* or leg* or knee* or ankle* or foot or feet or hip* or arm* or shoulder* or back* or sprain* or strain* or soft tissue* or muscle* or bone* or joint*) OR AB (skeletal muscle* or leg* or knee* or ankle* or foot or feet or hip* or arm* or shoulder* or back* or soft tissue* or muscle* or bone* or joint*) ) N3 (TI injur* OR AB injur*) ) AND ( (MH "Wounds and Injuries+") ) OR ( TI ( injur* or trauma* or rupture* or fracture* ) OR AB ( injur* or trauma* or rupture* or fracture* ) ) AND ( (MH "Physical Education and Training+") ) OR (MH "Preventive Health Care+") OR ( TI (prevent* or stretch* or training or protect* or precaution* ) OR AB ( prevent* or stretch* or training or protect* or precaution* ) )

**Cochrane Library**

ID Search

#1 MeSH descriptor: [Military Personnel] explode all trees

#2 MeSH descriptor: [Military Medicine] explode all trees

#3 MeSH descriptor: [Naval Medicine] explode all trees

#4 militar* or armed force* or air force* or army or armies or infantr* or soldier* or warfighter* or naval or navy:ti,ab,kw (Word variations have been searched)

#5 #1 or #2 or #3 or #4

#6 MeSH descriptor: [Musculoskeletal System] explode all trees

#7 MeSH descriptor: [Musculoskeletal Pain] explode all trees

#8 MeSH descriptor: [Sprains and Strains] explode all trees

#9 MeSH descriptor: [Cumulative Trauma Disorders] explode all trees

#10 MeSH descriptor: [Athletic Injuries] explode all trees

#11 MeSH descriptor: [Leg Injuries] explode all trees

#12 MeSH descriptor: [Hip Injuries] explode all trees

#13 MeSH descriptor: [Extremities] explode all trees and with qualifier(s): [Injuries - IN]

#14 MeSH descriptor: [Arm Injuries] explode all trees

#15 MeSH descriptor: [Back Injuries] explode all trees

#16 MeSH descriptor: [Soft Tissue Injuries] explode all trees

#17 MeSH descriptor: [Exercise] explode all trees

#18 MeSH descriptor: [Sports] explode all trees

#19 MeSH descriptor: [Track and Field] explode all trees

#20 musculoskeletal or musculo-skeletal or overus* or overtrain* or overexertion* or over-exertion* or overstrain* or excessive training or cumulative trauma or sprain* or strain*:ti,ab,kw (Word variations have been searched)

#21 (skeletal muscle* or leg* or knee* or ankle* or foot or feet or hip* or arm* or shoulder* or back* or soft tissue* or muscle* or bone* or joint*) near/3 injur*:ti,ab,kw (Word variations have been searched)

#22 #6 or #7 or #8 or #9 or #10 or #11 or #12 or #13 or #14 or #15 or #16 or #17 or #18 or #19 or #20 or #21

#23 MeSH descriptor: [Wounds and Injuries] explode all trees

#24 injur* or trauma* or rupture* or fracture*:ti,ab,kw (Word variations have been searched)

#25 #23 or #24

#26 MeSH descriptor: [Physical Education and Training] explode all trees

#27 prevent* or stretch* or training or protect* or precaution*:ti,ab,kw (Word variations have been searched)

#28 MeSH descriptor: [Accident Prevention] explode all trees

#29 MeSH descriptor: [Preventive Medicine] explode all trees

#30 #26 or #27 or #28 or #29

#31 #5 and #22 and #25 and #30

**WHO trial registry:**

Basic search: military AND injury AND prevention

Advanced search: militar* or armed force* or air force* or army or armies or infantr* or soldier* or warfighter* or naval or navy AND Prevention

**Opengrey:**

(military OR armed force) AND injuries

[**Greylit**](http://www.greylit.org/)**:**

(military OR armed force) AND injuries

**SPORTDISCUS (via Ebsco):**

Military Personnel OR Military Medicine OR ( TI ( militar* or armed force* or air force* or army or armies or infantr* or soldier* or warfighter* or naval or navy ) OR AB ( militar* or armed force* or air force* or army or armies or infantr* or soldier* or warfighter* or naval or navy ) )

AND

Musculoskeletal System OR ( Sprains and Strains ) OR Athletic Injuries OR Leg Injuries OR Arm Injuries OR Back Injuries OR Soft Tissue Injuries OR ( Track and Field ) OR ( TI ( musculoskeletal or musculo-skeletal or overus* or overtrain* or overexertion* or over-exertion* or overstrain* or excessive training or cumulative trauma or sprain* or strain* ) OR AB ( musculoskeletal or musculo-skeletal or overus* or overtrain* or overexertion* or over-exertion* or overstrain* or excessive training or cumulative trauma or sprain* or strain* ) ) OR ( TI ( exercise injur* OR sport injur* ) OR AB ( exercise injur* OR sport injur* ) ) OR ( ( TI ( skeletal muscle* or leg* or knee* or ankle* or foot or feet or hip* or arm* or shoulder* or back* or sprain* or strain* or soft tissue* or muscle* or bone* or joint* ) OR AB ( skeletal muscle* or leg* or knee* or ankle* or foot or feet or hip* or arm* or shoulder* or back* or sprain* or strain* or soft tissue* or muscle* or bone* or joint* ) ) AND ( TI ( injur* OR AB injur* ) OR AB ( injur* OR AB injur* ) ) )

AND

( Wounds and Injuries ) OR ( TI ( injur* or trauma* or rupture* or fracture* ) OR AB ( injur* or trauma* or rupture* or fracture* ) )

AND

( Physical Education and Training ) OR Preventive Health Care OR ( TI ( prevent* or stretch* or training or protect* or precaution* ) OR AB ( prevent* or stretch* or training or protect* or precaution* ) )

Appendix B: Reason for excluded studies

| Study | Reason for exclusion |
| --- | --- |
| Albano 1998 | Study design does not meet the inclusion criteria. |
| Alricsson 2004 | Study design does not meet the inclusion criteria. |
| Grier 2015 | Study design does not meet the inclusion criteria. |
| Hall 2013 | No full text available. We contacted the authors, but did not receive response. |
| Hartig 1999 | Study design does not meet the inclusion criteria. |
| Hofstetter 2011 | Study design does not meet the inclusion criteria. |
| Knapik 2005 | Study design does not meet the inclusion criteria. |
| Knapik 2004 | Study design does not meet the inclusion criteria. |
| Knapik 2008 | Study design does not meet the inclusion criteria. |
| Larsson 2012 | Study design does not meet the inclusion criteria. |
| Luippold 2011 | Study design does not meet the inclusion criteria. |
| Mann 2002 | No full text available. We contacted the authors, but did not receive response. |
| Palmanovich 2016 | Language does not meet the inclusion criteria (article in Hebrew). |
| Popovich 2000 | Study design does not meet the inclusion criteria. |
| Roos 2015 | Study design does not meet the inclusion criteria. |
| Sovelius 2006 | Study design does not meet the inclusion criteria. |
| Walker 2011 | Study design does not meet the inclusion criteria. |
| Woodruff 1994) | Study design does not meet the inclusion criteria. |

**References to studies excluded from this review**

1. Albano, J. J. and J. B. Stanford (1998). "Prevention of minor neck injuries in F-16 pilots." Aviation Space and Environmental Medicine 69(12): 1193-1199.
2. Alricsson, M., et al. (2004). "Neck muscle strength and endurance in fighter pilots: effects of a supervised training program." Aviat Space Environ Med 75(1): 23-28.
3. Grier, T., et al. (2015). "The effects of cross-training on fitness and injury in women." US Army Med Dep J: 33-41.
4. Hall, N., et al. (2013). "Does a structured neuromuscular training program reduce the incidence of lower limb injuries in NZDF army recruits?" Journal of Science and Medicine in Sport 16: e17.
5. Hartig, D. E. and J. M. Henderson (1999). "Increasing hamstring flexibility decreases lower extremity overuse injuries in military basic trainees." Am J Sports Med 27(2): 173-176.
6. Hofstetter, M. C., et al. (2011). "Effects of a 7-week outdoor circuit training program on Swiss Army recruits." J Strength Cond Res 26(12): 3418-3425.
7. Knapik, J., et al. (2005). "Evaluation of a standardized physical training program for basic combat training." J Strength Cond Res 19(2): 246-253.
8. Knapik, J. J., et al. (2004). "Influence of an injury reduction program on injury and fitness outcomes among soldiers." Inj Prev 10(1): 37-42.
9. Knapik, J. J., et al. (2008). "Parachute ankle brace and extrinsic injury risk factors during parachuting." Aviat Space Environ Med 79(4): 408-415.
10. Larsson, H., et al. (2012). "Influence of the implementation of a comprehensive intervention programme on premature discharge outcomes from military training." Work 42(2): 241-251.
11. Luippold, R. S., et al. (2011). "Effectiveness of an external ankle brace in reducing parachuting-related ankle injuries." Injury Prevention (1353-8047) 17(1): 58-61.
12. Mann, G., et al. (2002). Preventive effects of an on-shoe brace on ankle sprains in infantry. In Nyska, M. (ed.), The unstable ankle, Champaign, Ill., Human Kinetics, c2002, p.292-305.;.
13. Palmanovich, E., et al. (2016). "[A NOVEL GENDER-SPECIFIC VEST FOR FEMALE INFANTRY RECRUITS DOES NOT REDUCE THE INCIDENCE OF OVERUSE PAIN SYNDROMES: A PROSPECTIVE RANDOMIZED STUDY AMONG 240 RECRUITS]." Harefuah 155(6): 357-359, 386.
14. Popovich, R. M., et al. (2000). "Effect of rest from running on overuse injuries in army basic training." Am J Prev Med 18(3 Suppl): 147-155.
15. Roos, L., et al. (2015). "Adapted marching distances and physical training decrease recruits' injuries and attrition." Mil Med 180(3): 329-336.
16. Sovelius, R., et al. (2006). "Trampoline exercise vs. strength training to reduce neck strain in fighter pilots." Aviat Space Environ Med 77(1): 20-25.
17. Walker, T. B., et al. (2011). "Adaptations to a new physical training program in the combat controller training pipeline." J Spec Oper Med 11(2): 37-44.
18. Woodruff, S. I., et al. (1994). "The U.S. Navy Healthy Back Program: effect on back knowledge among recruits." Mil Med 159(7): 475-484.

Appendix C: Characteristics of included studies

Amako 2003

| Methods | Cluster - Randomised controlled trial |
| --- | --- |
| Participants | 18-25 years old, members of the 10st Educational Battalion, Camp Beppu, JGSDF, 901 healthy male subjects |
| Interventions | Stretching program  Control group (non-stretching group) |
| Outcomes | 1. The overall injury rate over the study period (percentage of recruits injured),  2. The location-specific injury rate  3. The monthly injury rate (percentage of recruits injured per month). |

Risk of bias table

| **Bias** | **Authors’ judgement** | **Support for judgement** |
| --- | --- | --- |
| Random sequence generation (selection bias) | Unclear risk | Quote: ''Allocation to interventions took place after randomisation.''  Comment: method of randomisation procedure not described.  There is insufficient information to allow judgement. |
| Allocation concealment (selection bias) | High risk | Quote: ''In 1996, one company consisting of 82 recruits participated in this program with their company commanders approval. In 1997, two companies each consisting of 128 recruits participated with their commanders approval. Again in 1998, two companies each consisting of 90 recruits participated with their commanders approval. The nonparticipating companies in the battalion comprised the control group.''  Comment: the method contains any other explicitly unconcealed procedure. |
| Blinding of participants and personnel (performance bias) | High risk | Quote: ''No blinding of personnel"  Quote: ''Participating companies in the battalion comprise the intervention groups. The nonparticipating companies in the battalion comprised the control group: not blinded for the commander (personnel).''  Comment: No blinding of personnel and the outcome is likely to be influenced by lack of blinding. Blinding of participants is unclear. |
| Blinding of outcome assessment (detection bias) | Unclear risk | Comment: The study did not address this outcome. |
| Incomplete outcome data (attrition bias) | High risk | Comment: injury rates in section results in the article are different than the reported injury rates in table 1.  Reported in section 'results': the injury rate in the stretching group was 11.2% (58 injuries). Table 1 shows the same number of injuries ( 58) but a different injury rate (11.2%).  Reported in section 'results': The injury rate in the non-stretching group was slightly higher at 14.1% (56 injuries).Table 1 shows the same number of injuries ( 56) but a different injury rate (14.6%). |
| Selective reporting (reporting bias) | Unclear risk | Comment: insufficient information to permit judgement of Low risk or High risk. Moreover, the study protocol is not available. |
| Other bias | Unclear risk | Comment: no adjustment for clustering. Moreover, demographic characteristic table is not presented in the article. |

Brushoj 2008

| Methods | Cluster - Randomised controlled trial |
| --- | --- |
| Participants | 1020 army conscripts were included consecutively as they were enrolled in the Royal Danish Life Guards. |
| Interventions | 1. The prevention training program (PRE)  2. Placebo training program (PLA) |
| Outcomes | 1. Primary outcome: overuse knee injuries (patellofemoral pain syndrome, iliotibial band friction syndrome, jumpers knee) or medial tibial stress syndrome.  2. Secondary outcome: any injury to the lower extremity |

Risk of bias table

| **Bias** | **Authors’ judgement** | **Support for judgement** |
| --- | --- | --- |
| Random sequence generation (selection bias) | Unclear risk | Quote: ''The conscripts were randomly divided by personal registration number into 8 companies each consisting of 3 platoons, with 2 companies/6 platoons beginning their training every fourth month.  Quote: ''The randomization was performed by the head nurse, who otherwise did not participate in the study.''  Quote: ''In addition, cluster randomisation was performed on the platoons.''  Comment: insufficient information to allow judgement. |
| Allocation concealment (selection bias) | Unclear risk | Quote: ''The randomization was performed by the head nurse, who otherwise did not participate in the study.''  Quote: ''The recruits were randomly divided (by personal registration number) into 8 companies.''  Comment: insufficient information to allow judgement. |
| Blinding of participants and personnel (performance bias) | Low risk | Quote: '' The recruits did not know which of the training programs was being tested.''  Comment: however, blinding is not possible because of the  visibility of the intervention. The review authors judge that the outcome is not likely to be influenced by lack of blinding. |
| Blinding of outcome assessment (detection bias) | Low risk | Quote: '' All subjects with knee pain or shin pain were examined every second week by one of the authors (C.B.) who was blinded to training group allocation. Before their examination, the patients were informed by the nurse not to reveal what exercise group they were allocated to.'' Comment: blinding of outcome assessment ensured, and  unlikely that the blinding could have been broken. |
| Incomplete outcome data (attrition bias) | Low risk | Quote: '' The time to onset of overuse knee injuries and shin pain is shown in Table 5. As there was no difference in injury incidence between the PRE and PLA groups, we performed no further analysis on the time to onset of injury between the groups.''  Comment: there were 10 recruits excluded because they failed to appear and 23 recruits dropout. However, an intention to treat analysis was performed. |
| Selective reporting (reporting bias) | Unclear risk | Comment: the study protocol is not available. |
| Other bias | Unclear risk | Comment: No adjustment for clustering performed. |

Carow 2014

| Methods | Cluster- Randomised controlled trial |
| --- | --- |
| Participants | College freshmen from the incoming class of new cadets at the USMA during the summer of 2010. The 1374 new cadets (1070 men, 243 women). |
| Interventions | 1. DIME cadre supervised (DCS)  2. DIME expert supervised (DES)  3. Active warm-up (AWU) |
| Outcomes | Cumulative incidence of musculoskeletal injury to the lower extremities during 3 time frames (CBT, academic year, and CBT and academic year combined) and  risk of acute traumatic knee-joint injury and ACL injury. |

Risk of bias table

| **Bias** | **Authors’ judgement** | **Support for judgement** |
| --- | --- | --- |
| Random sequence generation (selection bias) | Unclear risk | Comment: insufficient information to allow judgement. |
| Allocation concealment (selection bias) | Unclear risk | Comment: method of concealment is not described. Insufficient information to allow judgement. |
| Blinding of participants and personnel (performance bias) | High risk | Quote: '' The PTs did not ask cadets which group they were assigned to during their evaluations and were blinded to group assignment.''  Comment: not mentioned whether the supervision of upper-class cadet instructors were blinded or not. Cadets knew which group they were assigned to and outcome could be influenced by lack of blinding. |
| Blinding of outcome assessment (detection bias) | Low risk | Comment: the orthopedic surgeons and primary care physicians who evaluated the cadets were also blinded to group assignment. |
| Incomplete outcome data (attrition bias) | Low risk | Comment: no missing data outcome. |
| Selective reporting (reporting bias) | Unclear risk | Comment: the study protocol is not available. |
| Other bias | Unclear risk | Comment: no adjustment for clustering performed. |

Childs 2010

| Methods | Cluster - Randomised controlled trial. |
| --- | --- |
| Participants | The study participants were soldiers with a mean age of 22.9 years (SD=4.7, range=18-35) for whom complete injury data were available for analysis (n=1,141). Male and female. |
| Interventions | CSEP (core stabilization exercise program)  TEP (traditional exercise program) |
| Outcomes | Musculoskeletal injuries, non-musculoskeletal injuries and work restriction due to musculoskeletal injuries. |

Risk of bias table

| **Bias** | **Authors’ judgement** | **Support for judgement** |
| --- | --- | --- |
| Random sequence generation (selection bias) | Low risk | Quote: ''The randomization schedule was prepared by computer before recruitment began and was balanced to ensure equal allocation to both conditions after 20 companies were recruited.''  Comment: randomisation was done using a computer random number generator. |
| Allocation concealment (selection bias) | Low risk | Quote: ''The randomization schedule was prepared by computer before recruitment began and was balanced to ensure equal allocation to both conditions after 20 companies were recruited.'' |
| Blinding of participants and personnel (performance bias) | High risk | Quote: ''It was not possible to prevent soldiers from being aware of their group assignments because they actively participated in their randomly assigned training programs. Given the nature of the outcomes of this study, no blinding could possibly lead to performance bias.  Quote: ''However, APFT scores (the Army Physical Fitness Test) were collected by drill instructors according to the standard testing procedures. The drill instructors were not formally involved with the study other than within the context of the usual training environment.''  Comment: no blinding or incomplete blinding and the review authors judge that the outcome is likely to be influenced by lack of blinding. |
| Blinding of outcome assessment (detection bias) | Low risk | Quote: ''Study-related measures were collected before training and 12 weeks later, when training was completed, by study personnel who were unaware of the randomization assignments.''  Comment: blinding of outcome assessment ensured, and unlikely that the blinding could have been broken. |
| Incomplete outcome data (attrition bias) | High risk | Quote: ''Soldiers with missing data were excluded because the purpose of this study was to determine the impact of a CSEP among soldiers who completed the full training period. Complete profile data were available for 1,141 (27.7%) of the randomized soldiers because of inconsistent reporting of profiles (Fig. 2).''  Comment: however, the rates of reporting were similar between the groups.  Comment: it is unclear whether the Missing Profile Data contains MSIs. The amount of missings is large and the reason of missings unclear. |
| Selective reporting (reporting bias) | Low risk | Comment: the study protocol is available^a^ and all of the study s pre-specified (primary and secondary) outcomes that are of interest in the review have been reported in the pre-specified way. |
| Other bias | Unclear risk | Quote: ''A limitation of this study was the inconsistent reporting of injuries during training. However, the rates of reporting were similar between the groups.''  Comment: the reasons of missings are unclear and could possibly be related to MSIs.  Comment: no adjustment for clustering. |

^a^(https://clinicaltrials.gov/ct2/show/record/NCT00373009?term=NCT00373009&rank=1)

Coppack 2011

| Methods | Cluster - Randomised controlled trial. |
| --- | --- |
| Participants | All British Army recruits who enlisted at the Army Training Centre (Pirbright, UK) between July 2006 and February 2007 and passed the entry medical examination were invited to participate in the study (n = 1502). |
| Interventions | 1. AKP (anterior knee pain) prevention training program (PTP)  2. Control program |
| Outcomes | 1. Primary: an incident case of overuse AKP occurring during the 14-week training period.  2. Secondary: occupational endpoints of each participant: successful completion, medical discharge (MD), discharge as of right (DAOR: a voluntary discharge at the request of the recruit), unfit for army service (UFAS: recruits incapable of meeting the training standards), backsquadding (recruits held back in training), and other (withdrawal from training for all other reasons). |

Risk of bias table

| **Bias** | **Authors’ judgement** | **Support for judgement** |
| --- | --- | --- |
| Random sequence generation (selection bias) | Low risk | Quote: ''Each of the 50 troops (clusters) (average number of recruits [range]: 41 [22-48]) were randomly assigned to 1 of 2 groups: an AKP prevention training program (PTP) or a control program. A simple randomization procedure based on a computer-generated table of random numbers was used to allocate the intervention, and an external administrator provided the group assignment.''  Comment: randomization procedure using a computer random number generator. |
| Allocation concealment (selection bias) | Unclear risk | Quote: ''An external administrator provided the group assignment.''  Comment: no method was described to conceal allocation.  Insufficient information to allow judgement. |
| Blinding of participants and personnel (performance bias) | High risk | Quote: ''An attempt was made to blind participants, but given the physical nature of the intervention, we refrain from calling this a double-blinded study.''  Quote: ''Participant blinding was attempted through the application of dummy warm-up exercises for control group participants.''  Comment: filling in the time that is used for the intervention group for the prevention program with a dummy warm-up doesn't blind the participants and personnel completely. After all, the intervention is visible for everyone and can't be blinded. Moreover, the article doesn't describe whether personnel was blinded or not to the intervention. Given the nature of the intervention and the fact that some of the personnel were trained to guide the intervention, blinding was not done.  Comment: no blinding or incomplete blinding could have been effected the outcome (performance bias), given the nature of the outcome (AKP). |
| Blinding of outcome assessment (detection bias) | High risk | Quote: ''Physicians diagnosing AKP cases were blinded. Participants in different groups did not attend concurrent physical training sessions and were instructed not to reveal  information about sessions to the AKP outcome assessor (physiotherapist).''  Quote: ''Five experienced physiotherapists who were blinded to group assignment made a diagnosis of AKP.''  Blinding failed: Quote: ''Blinding of outcome assessors was not completely successful. In 33% (6 in the intervention group and 9 in the control group) of all AKP cases, the trained observer responded that he or she was aware of participant allocation. In procedural intervention trials this can account for an overestimation of effect by up to 17%.''  Comment: Blinding of outcome assessment, but likely that the blinding could have been broken, and the outcome measurement is likely to be influenced by lack of blinding. |
| Incomplete outcome data (attrition bias) | Low risk | Quote: ''No individuals were lost to follow-up'' Comment: no missing outcome data. |
| Selective reporting (reporting bias) | Low risk | Comment: the study protocol is available and all pre-specified (primary and secondary) outcomes that are of interest in the review have been reported in the pre-specified way. |
| Other bias | High risk | Quote: ''Unfortunately, because of military operational commitments, data collection was stopped early, and the estimate of the effect lacks precision. However, the study was still adequately powered because the effect we saw was larger than hypothesized. It is possible that there was some censorship bias because of more participants from the control group being medically discharged; if individuals who were medically discharged were also more likely to develop AKP, then the effect would have been underestimated.''  Comment: deviation from the study protocol. There was probably not enough time for occurrence of the event (to use cox proportional hazard regression; assumption): there was no follow-up beyond 14 weeks, and so no conclusions can be drawn about the long-term benefits of the intervention.  Quote: ''The total exposure time was 2034 recruit training months in the intervention group and 1824 recruit training months in the control group.''  Comment: follow-up time was different between the groups. |

Dijksma 2019

| Methods | Cluster - Randomised controlled pilot study |
| --- | --- |
| Participants | 64 recruits undergoing 23 weeks initial military training with the Air Manoeuvre Brigade |
| Interventions | 1. Agility training (AT)  2, Usual training program |
| Outcomes | Secondary: Withdrawal from initial military training due to injuries |
| Notes | Of the 36 planned AT sessions, 26 (72%) occurred. |

Risk of bias table

| **Bias** | **Authors’ judgement** | **Support for judgement** |
| --- | --- | --- |
| Random sequence generation (selection bias) | Low risk | Quote: "This was a cluster randomised controlled pilot study of four BMT classes. Human Resources divided these before BMT into balanced classes based on age, prior education and future military position after graduation. Classes receive physical training in pairs. Independent from the grouping, we used cluster randomisation to assign the intervention to two of the four classes, which formed the intervention (AT) and control groups (CG)." |
| Allocation concealment (selection bias) | Unclear risk | Quote: "An independent study assistant who drew lots performed randomisation"  Comment: however, the authors did not describe the method of concealment. Thus, no judgement can be made regarding selection bias. |
| Blinding of participants and personnel (performance bias) | High risk | Quote: "All participants were briefed on the study objectives in week 1 of the BMT by the lead author (ID)" Comment: the intervention (agility training) is an intervention which is not possible for blinding (visible intervention). It can be assumed that the sports instructors were not blinded for the intervention. Comment: no blinding of participants could have been influenced the outcome (performance bias) |
| Blinding of outcome assessment (detection bias) | High risk | Quote: "The platoon commander registered dropout-rates and reason for dropout at the moment the recruit was dismissed from the training program."  Comment: Detection bias could have occurred. |
| Incomplete outcome data (attrition bias) | Low risk | Comment: no incomplete outcome data |
| Selective reporting (reporting bias) | Unclear risk | Comment: the study protocol is not available. |
| Other bias | Low risk | Quote: "Intention-to-treat analysis of the primary outcome body control and CODS included paired students t-tests to estimate within-group changes, and linear regression analysis with adjustment for the score at T0 to estimate between-group differences. We performed a multilevel analysis to assess the need to account for clustering. This analysis showed no statistically significant intra-cluster correlation coefficient, and the results were comparable to those of the ordinary least square regression analysis." |

Goodall 2012

| Methods | Cluster - Randomised controlled trial |
| --- | --- |
| Participants | Army recruits, newly enlisted into the Australian Army and undertaking basic training at ARTC. 732 male and 47 female army recruits from the Australian Army Recruit Training Centre. |
| Interventions | 1. 80-day basic training program/ normal physical training (control group)  2. 80-day combat Agility Program (CAP) (intervention group) |
| Outcomes | The incidence rates over 12 weeks of basic training for (1) knee and ankle injuries, (2) knee and ankle ligament injuries, and (3) lower limb injuries. |

Risk of bias table

| **Bias** | **Authors’ judgement** | **Support for judgement** |
| --- | --- | --- |
| Random sequence generation (selection bias) | Low risk | Quote: ''The allocations were prepared prior to the commencement of the study, using the planned blocking procedure and a published random number table and randomisation procedure (Pocock, 1983, pp. 73 74), starting at row 3, column 6, reading to the right and allocating the first cohort in each block to the intervention group for a digit in the range 0 4 and to the control group for a digit in the range 5 9.''  Comment: randomization using a random number table. |
| Allocation concealment (selection bias) | Low risk | Quote: ''Based on the study design, the 10 cohorts of participants were allocated to IG or CG, using a concealed, blocked (stratified), random allocation procedure.''  Quote: ''When each new cohort of participants entered the study following informed consent to participate, the next envelope in the numbered sequence of envelopes was opened by the principal researcher, revealing the group to which the cohort would be assigned (IG or CG)'' Comment: allocation of concealment using sequentially numbered, opaque, sealed envelopes. |
| Blinding of participants and personnel (performance bias) | Unclear risk | Comment: blinding of participants and personnel not mentioned in the article. The intervention is visible to the personnel and the participant, thus blinding could not have been done. An injury was defined in this study as physical harm to the musculoskeletal structures of the body for which health care was required and which prohibited the recruit from performing normal military training, free of any injury symptoms, for at least one day. That means that the outcome is likely to be influenced by lack of blinding. But still, there is insufficient information to allow judgement. |
| Blinding of outcome assessment (detection bias) | Low risk | Quote: ''The ARTC health staff who recorded the injuries were blinded to the group allocation of participants.'' Comment: blinding of outcome assessment ensured, and unlikely that the blinding could have been broken. |
| Incomplete outcome data (attrition bias) | High risk | Quote: ''IG lost-to follow up:n=33 (9%) (= discharged from the Army), n= 46 (12%) (discontinued intervention; failed training and 'backsquadded' to another cohort but injuries reported.  CG lost-to follow up:n=55 (11%) (= discharged from the Army), n= 39 (8%) (discontinued intervention; failed training and 'backsquadded' to another cohort but injuries reported.''  Quote: ''Intention to treat analysis performed with those excluded from the Army ( IG n=347; CG n= 432).''  Comment: However, participants who were discharged from the army prior to completing training were deemed lost to follow-up and removed from the analysis and reasons of discharge were not described. |
| Selective reporting (reporting bias) | Unclear risk | Quote: ''The trial was approved by the Australian Defence Human Research Ethics Committee, protocol number 464/06'' Comment: the study protocol is not available (not found). |
| Other bias | Unclear risk | Comment: baseline imbalances not reported. Not reported whether they adjust for clustering. Selection bias could bias the estimate of effect, given the fact that randomisation on cluster level with 10 clusters will not be enough to prevent selection bias. However, insufficient information to allow judgement. |

Marcinik 1987

| Methods | Randomised controlled trial |
| --- | --- |
| Participants | 1169 male recruits receiving 8-week Navy basic training at the Recruit Training Command, San Diego, California |
| Interventions | 1. Standard aerobic/calisthenic (A/CAL) program  2. Circuit weight training regimen performed in conjunction  3. Standard running program |
| Outcomes | 1. Primary: sprain and strain injuries incl. particular location of the S/S injury (e.g.. shoulder/arm, Knee/leg, ankle/foot, and lower back)  2. Secondary: the number of no march/no physical training CNM/NPT) days lost on account of Injury |
| Notes | In the standard aerobic/calisthenic (A/CAL) program group it's not clear what '10 minutes of flexibility and calistenic exercises' contains. Both groups received a running program, but the only difference is that one group received 10 minutes of flexibility and calistenic exercises while the other group received circuit weight training exercises. The question is whether the calistenic exercises are similar with weight training exercises. |

Risk of bias table

| **Bias** | **Authors’ judgement** | **Support for judgement** |
| --- | --- | --- |
| Random sequence generation (selection bias) | Unclear risk | Comment: No randomisation procedure described. Insufficient information to allow judgement. |
| Allocation concealment (selection bias) | Unclear risk | Comment: the study did not address this outcome. Insufficient information to allow judgement. |
| Blinding of participants and personnel (performance bias) | High risk | Comment: Impossible to blind the participants and personnel and the outcome is likely to be influenced because of the visibility of the intervention. |
| Blinding of outcome assessment (detection bias) | High risk | Comment: Outcome was done self-reported by medical vouchers; participants were not blinded and the outcome is likely to be influenced. |
| Incomplete outcome data (attrition bias) | Unclear risk | Comment: Insufficient reporting of attrition/exclusions to permit judgement. |
| Selective reporting (reporting bias) | Unclear risk | Comment: one or more outcomes of interest in the review are reported incompletely so that they cannot be entered in a meta-analysis; nothing reported about attrition rate, compliance and missing data; no baseline information of the two groups reported; it is unclear whether possible baseline differences influences the outcomes in terms of bias. |
| Other bias | Unclear risk | Comment: Insufficient information about several aspects (see above)  Comment: No adjustment for clustering |

Meijer 2016

| Methods | Cluster- Randomised controlled trial |
| --- | --- |
| Participants | Marine Corps recruits starting the 24 week initial training of the Navy Seals . Participants (> 18 years) who passed the medical, psychological, physical test and the Marines Selection Test are admitted to the class EMV MARNS 15/2. |
| Interventions | Core stability training  Control group, usual training program |
| Outcomes | Non-traumatic musculoskeletal injuries  Dropout  Adverse effects. |
| Notes | This the trial is still on-going. The whole trial is comprised of two initial navy Seal training periods EMV MARNS 15/2 & 16/1). The first period EMV MARNS 15/2 is now evaluated.  A weakness of this study is, that it was under-powered, so no significant conclusions can be reported. The trial findings are imprecise so the external validity remains low. |

Risk of bias table

| **Bias** | **Authors’ judgement** | **Support for judgement** |
| --- | --- | --- |
| Random sequence generation (selection bias) | Low risk | Quote: ''A computer-generated list of random numbers. was used to generate the random sequence.''  Comment: randomization procedure using a computer random number generator. |
| Allocation concealment (selection bias) | Low risk | Quote: ''The randomization, generation of the allocation sequence, enrolment of participants and assignment of participants to interventions was performed by the data manager of the Fieldlab, not informed for demographic, functional movement screen and sport history data.  Allocation was implemented by the data-manager of the Fieldlab and allocation was concealed for the participants and all other involved until the start of the initial training.''  Quote: ''Allocation concealment was maintained for the Medical service and management of the MOC (outcome registration)''  Comment: web-based randomisation: central allocation.  Concealment of allocation has been ensured. |
| Blinding of participants and personnel (performance bias) | High risk | Personnel: Quote: '' In this non-pharmacological trial later blinding was not possible for physical trainers, who implemented the core stability training.''  Participants: Quote: '' The blinding for allocation is realized by creating separate classes for the intervention group and control group. Both groups were extensively informed over the core stability research taking place and asked for their consent.''  Comment: No blinding or incomplete blinding and the review authors judge that the outcome is likely to be influenced by lack of blinding (see outcome type) |
| Blinding of outcome assessment (detection bias) | Low risk | Quote: ''The outcome assessors, doctors and management of the MOC, were blinded for the allocation and the results of the FMS test. The FMS raters are blinded for the allocation. The physical trainers as well as the management of the MOC are blinded for the measurements of physical fitness, demographic variables, the FMS score and the sport-history items. The data-analyst was blinded until the entire analysis was completed.''  Comment: Blinding of outcome assessment ensured, and unlikely that the blinding could have been broken. |
| Incomplete outcome data (attrition bias) | Low risk | Comment: No incomplete outcome data. |
| Selective reporting (reporting bias) | High risk | Comment: The study protocol is available.  - The study protocol: the Hazard ratio. The incidence density for injuries of the intervention and the control group are analyzed  - The original article: The relative risk for injury (as registered by the Medical Service) from intervention versus control is.  The outcome measures are different in the protocol and the original article. |
| Other bias | Unclear risk | Comment: No adjustment for clustering. |

Parkkari 2011

| Methods | Cluster- Randomised controlled trial |
| --- | --- |
| Participants | Male conscripts from four companies of one brigade (Pori Brigade, Säkylä, Finland) in the Finnish Defence Forces. The Pori Brigade is a typical Finnish garrison, and the chosen companies formed a representative sample of conscripts. (n=2,025 conscripts. In flow chart: randomised 4 companies; n=1037 conscripts) |
| Interventions | 1. Neuromuscular training combined with Injury prevention counselling  2. Control group (usual training program) |
| Outcomes | 1. Acute lower- or upper-limb injury that occurred during the 6-month military service  2. Limited duty days. |

Risk of bias table

| **Bias** | **Authors’ judgement** | **Support for judgement** |
| --- | --- | --- |
| Random sequence generation (selection bias) | Low risk | Quote: ''Usual care/randomisation into companies: annually, the conscripts of each age cohort are randomly assigned into the companies.  The four companies were randomised into two groups (two intervention companies and two control companies). Using the company as the unit of randomisation with a computer-generated randomisation programme, an independent statistician who had no information about the study subjects performed the randomisation of companies into the intervention and control groups for the July 2007 and January 2008 cohorts.''  Comment: randomization procedure using computer-generated randomisation program. |
| Allocation concealment (selection bias) | Unclear risk | Quote: ''Using the company as the unit of randomisation with a computer-generated randomisation programme, an independent statistician who had no information about the study subjects performed the randomisation of companies into the intervention and control groups for the July 2007 and January 2008 cohort.'' |
| Blinding of participants and personnel (performance bias) | High risk | Quote: ''Companies allocated to the intervention group were informed about the upcoming programme for preventing injuries. the impossibility of full double blinding in this type of study limit the strength of the conclusions''  Comment: No blinding of participants and personnel: this could have influenced the outcome (limited duty days) |
| Blinding of outcome assessment (detection bias) | Low risk | Quote: ''The randomisation phase, data collection and data  analysis were fully blinded, but for obvious reasons the young conscripts and exercise instructors could not be masked.'' |
| Incomplete outcome data (attrition bias) | Low risk | Quote: ''During the intervention, 61 participants were lost to follow-up for medical reasons: 14 were permanently discharged from military service, and 47 were temporarily discharged for at least 6 months.''  Quote: ''Additionally, two conscripts were lost to follow-up because of a missing patient record, and one conscript applied for postponement of service during the run-in period.''  Quote: ''After two weeks run-in period a lots of lost to  follow-up: Intervention group: Completed intervention at 180 days (n=338/501). Control group: Completed intervention at 180 days (n=300/467)''  Comment: All analyses were performed according to the intention-to-treat principle. Missing data have been imputed using appropriate methods. |
| Selective reporting (reporting bias) | High risk | Comment: Flow chart doesn't match with the numbers of randomised described in the text: 'methods': n=2,025 conscripts.  'flow chart': randomised 4 companies; n=1037 conscripts. ' During the intervention, 61 participants were lost to  follow-up': doesn't match either with the flow chart: flow chart shows: n=35 (intervention group); n=34 (control group). thus 69 lost to follow-up during the intervention  Protocol: The clinical trial identification number is NCT00595816. Slight difference --> The study protocol is  available: study protocol: back problems--> article: upper limb. |
| Other bias | Unclear risk | Comment: No adjustment for clustering.  Comment: Co-intervention not clear. Probably no equal treatment in the control group between the companies: The 2-month basic training period was followed by 4 months specific military training programme, depending on the company and service duration. During this 6 month period of service, the amount and intensity of physical training was maintained at approximately the same level in different companies. No timing mentioned for the last 4 months were the companies trained with a different scheme. |

Pope 1998

| Methods | Cluster- Randomised controlled trial |
| --- | --- |
| Participants | 1093 male Australian Army recruits, aged between 17 and 35 years, who undertook recruit training between September 1992 and May 1993 in the Australian Army's 1st Recruit Training Battalion, situated at Kapooka, in rural New South Wales. |
| Interventions | 1. Calf muscle stretching compared  2. Non-calf muscle stretching. |
| Outcomes | Lower limb musculoskeletal injuries:  1. tendo-achilles lesions  2. lateral ankle sprains  3. stress fractures of the foot or tibia  4. periostitis of the tibia  5. anterior tibial compartment pressure syndrome (diagnosis supported later by measured compartment pressures of more than 15 mm Hg). |

Risk of bias table

| **Bias** | **Authors’ judgement** | **Support for judgement** |
| --- | --- | --- |
| Random sequence generation (selection bias) | Low risk | Quote: ''Randomisation to the platoons: Those recruits who consented to participate were allocated to stretch and control groups using a blocked, quasi-random allocation procedure. As male recruits arrived at Kapooka they were assigned by Army administrative staff to one of two platoons on the basis of surnames. Recruits with surnames commencing with the same letter were equally split between the two platoons. In addition, where possible, recruits with the same surname were allocated to alternate platoons.''  Quote: ''Randomisation of the interventions to the platoons: Pairs of platoons were then randomly allocated to control and stretch groups for this study.''  Comment: the overall allocation procedure is thus referred to here as 'quasi-random'. The investigators describe a random component in the sequence generation process. |
| Allocation concealment (selection bias) | Low risk | Quote: ''No other conditions influenced allocation to platoons (so only the surnames, but this is not something that the participants or personnel could have influenced). All allocation procedures to this point were conducted by administrative staff at Kapooka without regard for the research to be conducted.''  Comment: The size of the blocks were NOT small (26) (this, no high predictability of allocation). |
| Blinding of participants and personnel (performance bias) | Low risk | Participants: Quote: ''To partly mask the intervention, recruits were informed of the study in general terms only. They were not told which muscle group and injuries the researchers were investigating.''  Personnel: not described.  Comment: But the outcome is not likely to be influenced in case of no blinding or lack of blinding, because of the nature of the outcome (see outcomes above). |
| Blinding of outcome assessment (detection bias) | Unclear risk | Comment: Blinding of outcome assessors not described, but the outcome is likely to be influenced in case of no blinding or lack of blinding, because of the nature of the outcome no blinding could have been influenced the outcome. |
| Incomplete outcome data (attrition bias) | High risk | -Quote: ''162 (15 per cent) were discharged or backsquadded before the end of the training program, or before they experienced one of the five injuries of interest (Stretch group n= 98; control group n=64). Further 48 subjects (4 per cent), all from the control group, withdrew from the study; most withdrawals occurred at the end of the first half of the training program.''  Comment: However, reasons were not reported.  Comment: High amount of lost to follow-up  Comment: No imputation technique conducted |
| Selective reporting (reporting bias) | Unclear risk | Comment: the study protocol is not available. |
| Other bias | High risk | Comment: However, the exact method of stretching and the muscle groups to be stretched varied according to the instructors' preferences. The groups are probably not equally treated.  Quote: ''Medical assistants or nursing staff. When the injury was more than trivial, or when the recruit was unable to resume full duties without signs or symptoms within three days, these staff directed injured recruits to the regimental medical officer as standard procedure. But the researchers approached all medical assistants and nursing staff and reinforced the need to refer to the regimental medical officer all lower limb musculoskeletal injuries anyway, regardless of 'more than trivial, or loss of duty days'.''  Comment: Information bias:  Comment: No baseline differences reported and no adjustment for clustering |

Pope 2000

| Methods | Cluster- Randomised controlled trial |
| --- | --- |
| Participants | Male recruits entering the training program between January and December 1994 at the Australian Army s 1st Recruit Training Battalion, situated at Kapooka, in rural New South Wales. |
| Interventions | 1. Pre-exercise stretching compared  2. No stretching, control group |
| Outcomes | Any lower-limb injury that prevented the subject from resuming full duties, free of signs or symptoms, within 3 d. |

Risk of bias table

| **Bias** | **Authors’ judgement** | **Support for judgement** |
| --- | --- | --- |
| Random sequence generation (selection bias) | Low risk | Quote: ''Using a blocked, stratified, random allocation procedure. As the male recruits arrived at Kapooka, they were assigned to platoons on the basis of surnames, by army administrative staff. Recruits with surnames commencing with the same letter were equally split between platoons. In addition, where possible, recruits with the same surname were allocated to different platoons.''  Quote: ''Pairs of platoons, formed as described above, were then randomly allocated to stretch or control groups (stretch group 19 platoons, 735 subjects; control group 20 platoons, 803 subjects), so that one platoon from every pair was allocated to each group.''  Comment: The investigators describe a random component in the sequence generation process. |
| Allocation concealment (selection bias) | Low risk | Quote: ''No other conditions influenced allocation to platoons (so only the surnames, but this is not something that the participants or personnel could have influenced). All allocation procedures to this point were conducted by administrative staff at Kapooka without regard for the research to be conducted.''  Comment: The size of the blocks were NOT small (39) (this, no high predictability of allocation). |
| Blinding of participants and personnel (performance bias) | High risk | Personnel: Quote: ''Physical Training Instructors (PTI) were assigned to platoon pairs, so that the platoons allocated to each group were matched for PTI.''  Comment: No blinding of the personnel.  Comment: blinding for participants not described, but because of the visibility of the intervention, blinding was not possible.  The outcome is likely to be influenced in case of no blinding or lack of blinding, because of the nature of the outcome (see outcomes above). |
| Blinding of outcome assessment (detection bias) | Low risk | Quote: ''The RMO, who was masked to patient allocation.'' |
| Incomplete outcome data (attrition bias) | Low risk | Quote: ''Data from recruits who were backsquadded (reassigned to a later platoon because they were having difficulty with some aspect of training) and recruits who withdrew consent to participate in the study were analyzed by intention to treat (6,25,32). This meant that monitoring of these recruits continued after backsquadding or withdrawal, so that injury and time in training data were included in the statistical analyses as if the recruits continued with their allocated treatment.''  Comment: Reasons of lost to follow up reported and injury data were still available for these subjects, so their data were analyzed by intention to treat. Imputation of missing data done with appropriate imputation technique: intention to treat analysis. |
| Selective reporting (reporting bias) | Unclear risk | Comment: the study protocol is not available. |
| Other bias | High risk | - Quote: ''The initial procedure used to allocate recruits to platoons was unlikely to produce systematic differences between control and stretch groups, and systematic differences were made even more unlikely by random allocation of platoons to control or stretch groups.'' Comment: No adjustment for clustering done.  - Information bias: Quote: ''Medical assistants or nursing staff. When the injury was more than trivial, or when the recruit was unable to resume full duties without signs or symptoms within three days, these staff directed injured recruits to the regimental medical officer as standard procedure. But the researchers approached all medical assistants and nursing staff and reinforced the need to refer to the regimental medical officer all lower limb musculoskeletal injuries anyway, regardless of 'more than trivial, or loss of duty days'.''  - Quote: ''However, the exact method of stretching and the muscle groups to be stretched varied according to the instructors' preferences.''  Comment: The groups are probably not equally treated. |

Rudzki 1997

| Methods | Cluster- Randomised controlled trial |
| --- | --- |
| Participants | Male recruits drawn from all over Australia at the 1st Recruit Training Battalion (1 RfB), Wagga Wagga, New South Wales, during March and April 1989 and ranged in age from 17 to 31 years, with an average age of 19.1 years. |
| Interventions | 1. Substituted a weighted march activity for all running periods in the physical training program  2. Standard recruit training program. |
| Outcomes | 1. (Musculoskeletal) injuries  2. Loss of duty days. |

Risk of bias table

| **Bias** | **Authors’ judgement** | **Support for judgement** |
| --- | --- | --- |
| Random sequence generation (selection bias) | Low risk | Quote: “Recruits were allocated into platoons of approximately 45 by staff. Recruiting units faxed the names of recruits who were scheduled to arrive later that day. The training clerk allotted names to a platoon on an "as-received basis," i.e., the names of recruits were entered into platoon nominal rolls as the faxes were received. Once a platoon quota was filled, the second platoon was raised. a platoon was randomly assigned to be either a Walk or a Run group by being drawn from a hat by the author, who was blind to the composition of the groups.''  Comment: The investigators describe a random component in the sequence generation process. |
| Allocation concealment (selection bias) | Low risk | Quote: ''Once a platoon quota was filled, the second platoon was raised. a platoon was randomly assigned to be either a Walk or a Run group by being drawn from a hat by the author, who was blind to the composition of the groups.''  Quote: ''The clerk allocating recruits was thus blinded to any personal characteristics of the recruits.'' |
| Blinding of participants and personnel (performance bias) | High risk | Comment: Not possible to blind the intervention because of the visibility of the intervention. Outcome is likely to be influenced by no blinding. |
| Blinding of outcome assessment (detection bias) | Unclear risk | Comment: The study did not address this outcome. |
| Incomplete outcome data (attrition bias) | Unclear risk | Quote: ''The study was designed to capture medical data and causes for administrative discharge were not collected. The number of administrative discharges and backsquadding was higher in the Walk group. and the reasons for this were not clear.''  Quote: ''There were more medical discharges in the Run group. but this difference was not statistically significant ( Chi2= 3.10. P = 0.07).''  Comment: Insufficient reporting of attrition/exclusions to permit judgement of Low risk or High risk (reasons for missing data not completely provided). Not clear whether participants after censoring were excluded or not. |
| Selective reporting (reporting bias) | Unclear risk | Comment: the study protocol is not available. |
| Other bias | High risk | Comment: Definition of 'injuries' not reported  Comment: Text in the article 'results' does not match with table 1: text;90 and 109 recorded injuries, table: 90;111 recorded injuries  Quote: ''It was unfortunate that the true exposure varied somewhat in the Run group, but this was beyond the control of the experimental design (or the investigator)''  Comment: possible no equal treatment across the platoons.  Comment: No adjustment for clustering. |

Sell 2016

| Methods | Block - Randomised controlled trial |
| --- | --- |
| Participants | 101st Airborne Division (Air Assault) Soldiers |
| Interventions | 1. ETAP (Eagle Tactical Athlete Program )  2. Usual, existing physical training program at the Army s 101st Airborne Division (Air Assault) |
| Outcomes | Preventable injuries ( Examples include internal derangement of the knee, patellar tendonitis, and sprains and strains of shoulder and upper arm) (non-preventable injuries: Examples of injuries that were not classified as preventable include concussions, fractures (e.g., humeral fractures, and nerve entrapment injuries were not included) |
| Notes | Assumption: Block randomisation is cluster randomisation (based on deployment schedules) |

Risk of bias table

| **Bias** | **Authors’ judgement** | **Support for judgement** |
| --- | --- | --- |
| Random sequence generation (selection bias) | Unclear risk | Quote: "A total of 2,280 Soldiers consented to participate and were block assigned to the experimental group (EXP) and the control group (CON)."  Comment: Block randomisation procedure unclear. |
| Allocation concealment (selection bias) | Unclear risk | Comment: Block schedule not reported: when the size of the blocks were small, it could lead to a high predictability of allocation. |
| Blinding of participants and personnel (performance bias) | High risk | Comment: Groups were not blinded. Personnel could not be blinded because of the visibility of the intervention.  Outcome is likely to be influenced. |
| Blinding of outcome assessment (detection bias) | Unclear risk | Comment: Not reported whether the outcome assessors were blinded or not. If not, the outcome is likely to be influenced.  Cochrane: The study did not address this outcome. |
| Incomplete outcome data (attrition bias) | High risk | Quote: "The final data set included the 1,136 Soldiers in the EXP and 584 Soldiers in the CON." Reasons of missing not reported.  Per protocol analysis EXPGroup(n=1,136) CONGroup(n=584)  -Comment: Cochrane: As-treated analysis done with substantial departure of the intervention received from that assigned at randomization; |
| Selective reporting (reporting bias) | Unclear risk | Comment: Study protocol not reported. |
| Other bias | Unclear risk | Comment: Baseline differences not reported  Comment: No adjustment for clustering. |

Sharma 2014

| Methods | Randomised controlled trial. |
| --- | --- |
| Participants | British Army recruits classified as at risk of developing medial tibial stress syndrome. |
| Interventions | 1. Supervised gait retraining, including exercises to increase neuromuscular control and flexibility (three sessions per week) and biofeedback enabling internalization of the foot balance variable (one session per week) with as co-intervention 26-wk basic military training regimen.  2. Control group: only 26-wk basic military training regimen. |
| Outcomes | Medial tibial stress syndrome incidence. |

Risk of bias table

| **Bias** | **Authors’ judgement** | **Support for judgement** |
| --- | --- | --- |
| Random sequence generation (selection bias) | Low risk | Comment: Participants were assigned to groups using a blocked randomization schedule, with the six possible permutations of a fixed block size of four containing two control and two intervention assignment selected at random to create the allocation sequence.  Size of block randomisation not small: 4, randomisation is not predictable. + fixed block size  Cochrane: The investigators describe a random component in the sequence generation process. |
| Allocation concealment (selection bias) | Low risk | Comment: The sequence was concealed from the investigator assigning participants using opaque sealed envelopes.  Cochrane: opaque, sealed envelopes. |
| Blinding of participants and personnel (performance bias) | High risk | Comment: Blinding not possible because of the visibility of the intervention.  Quote: "It is not possible to blind participants to a gait retraining intervention."  Cochrane: No blinding or incomplete blinding, and the outcome is likely to be influenced by lack of blinding. |
| Blinding of outcome assessment (detection bias) | Low risk | Comment: Diagnoses of medial tibial stress syndrome over the 26-wk regimen were made by physicians blinded to the group assignment. Clinical diagnoses were made at the Army Medical Centre by military physicians who were blinded to the group assignment. |
| Incomplete outcome data (attrition bias) | High risk | Comment: Per protocol analysis-->intervention group; lost to follow up n=13 (other injuries; 5, stress fracture; 1, transfer; 1, discharges; 3, administration discharge; 3)  Control group: lost to follow up=17 (other injuries; 8, lower back pain; 1, transfer; 2, discharges; 4, administration discharge; 3)  Analysed; IG: n-70 and CG; n=66  Cochrane: As-treated analysis done with substantial departure of the intervention received from that assigned at randomisation; |
| Selective reporting (reporting bias) | Unclear risk | Comment: Study protocol not published. |
| Other bias | High risk | Comment: The intervention involved supervised gait retraining, including exercises to increase neuromuscular control and flexibility (three sessions per week) and biofeedback enabling internalization of the foot balance variable (one session per week)--> effect of the intervention could be influenced by exercises to increase neuromuscular control and flexibility (three sessions per week) and biofeedback enabling internalization of the foot balance variable.  Quote: "The gait-retraining program included biofeedback on risk factors and a battery of exercises to improve neuromuscular condition. We suggest that the biofeedback component might be a key contributor to the injury reduction observed. Although there are no previous biofeedback studies on actual injury incidence to support this suggestion, there have been several studies examining the effects of biofeedback on risk factors."  Comment: Exercises neuromuscular control: "Participants were encouraged to practice these exercises in their own time and to focus on the quality of the movements to compensate for the gradual reduction in supervision." Compliance? Not measured.  Comment: Effects were adjusted for baseline value of the outcomes to account for chance imbalance at baseline. But baseline variables not reported. |

Appendix D: ‘Summary of findings’ table Modification of training programs, in order of mentioning in study article

| **Study** | **Intervention** | **Outcome** | **Relative effect, 95% Confidence Interval** |
| --- | --- | --- | --- |
| Meijer, 2016 [48] | core stability exercises | non-traumatic musculoskeletal injuries | RR=1.04, 95%CI 0.78-1.38 |
|  |  | withdrawal from training | RR=1.04, 95%CI 0.82-1.33 |
| Childs, 2010 [40] | core stabilization exercise program without sit-up training compared to a traditional exercise program with sit-up training | any type of musculoskeletal injury | RR=1.09, 95%CI 0.96-1.24 |
|  |  | limited duty days | Mean decrease of 1 day, p=0.919 |
| Sharma, 2014 [38] | supervised gait retraining program | medial tibial stress syndrome | HR=0.25, 95%CI 0.05-0.53 |
| Rudzki [49] | substitution of running training by weighted marching | number of injured recruits | RR=1.24, 95%CI 0.98-1.61 |
|  |  | number of lower limbs injured | RR=1.65 95%CI 1.21-2.25 |
|  |  | number of knee injuries | RR=2.14, 95%CI 1.21-3.79 |
| Carow, 2016 [39] | dynamic integrated movement enhancement warm-up | incidence of lower extremity injuries | RR=1.02, 95%CI 0.85-1.22 |
| Coppack, 2011 [41] | Anterior knee pain prevention program | Anterior knee pain | RR=0.27, 95%CI 0.14-0.54 |
|  |  | discharges for medical reasons | RR=0.12, 95%CI 0.04-0.39 |
| Goodall, 2013 [42] | balance and agility training | lower limb injury incidence | RR=1.25, 95%CI 0.97-1.53 |
| Brushoj, 2008 [43] | exercise program with muscular strengthening, coordination and flexibility | overuse injury to the knee or shin | RR=1.05, 95%CI 0.98-1.11 |
| Parkkari, 2011 [50] | Neuromuscular and balance training with injury prevention counselling | acute ankle injuries | HR=0.34, 95%CI 0.15-0.78 |
|  |  | less time lost due to injuries | HR=0.55, 95%CI 0.29-1.04 |
| Dijksma, 2019 [51] | Agility Training | attrition due to injuries | RR=0.32, 95%CI 0.12-0.85 |
| Marcinik, 1987 [37] | circuit weight training | sprain and strain injury incidence | RR=0.82, 95%CI 0.72-0.93 |
|  |  | number of no march/no physical training days lost on account of injury | 123 days in the intervention group versus 330 days in the control group, p<0.05 |
| Sell, 2016 [44] | a program including speed/agility/balance, muscular strength, interval running, power, and endurance training | of any type of musculoskeletal injury | RR=0.66, 95%CI 0.47-0.93 |
